# Bone Marrow Mesenchymal Stem Cells Inhibit Lipopolysaccharide-Induced Inflammatory Reactions in Macrophages and Endothelial Cells

**DOI:** 10.1155/2016/2631439

**Published:** 2016-01-26

**Authors:** Dequan Li, Cong Wang, Chuang Chi, Yuanyuan Wang, Jing Zhao, Jun Fang, Jingye Pan

**Affiliations:** ^1^Department of Traumatology Medicine, The First Affiliated Hospital of Wenzhou Medical University, Wenzhou, Zhejiang 325000, China; ^2^School of Pharmacy, Wenzhou Medical University, Wenzhou, Zhejiang 325000, China; ^3^Department of Cardiothoracic Surgery, The First Affiliated Hospital of Wenzhou Medical University, Wenzhou, Zhejiang 325000, China; ^4^Department of Intensive Care Unit, The First Affiliated Hospital of Wenzhou Medical University, Wenzhou, Zhejiang 325000, China

## Abstract

*Background*. Systemic inflammatory response syndrome (SIRS) accompanied by trauma can lead to multiple organ dysfunction syndrome (MODS) and even death. Early inhibition of the inflammation is necessary for damage control. Bone marrow mesenchymal stem cells (BMSCs), as a novel therapy modality, have been shown to reduce inflammatory responses in human and animal models.* Methods*. In this study, we used Western blot, quantitative PCR, and enzyme-linked immunosorbent assay (ELISA) to assess the activity of BMSCs to suppress the inflammation induced by lipopolysaccharide (LPS) in human umbilical cord endothelial cells (HUVECs) and alveolar macrophages.* Results*. Our results demonstrated that LPS caused an inflammatory response in alveolar macrophages and HUVECs, increased permeability of HUVEC, upregulated expression of toll-like receptor (TLR) 2, TLR4, phosphorylated p65, downregulated release of IL10, and promoted release of TNF-*α* in both cells. Coculture with BMSCs attenuated all of these activities induced by LPS in the two tested cell types.* Conclusions*. Together, our results demonstrate that BMSCs dosage dependently attenuates the inflammation damage of alveolar macrophages and HUVECs induced by LPS.

## 1. Introduction

Traumatic injury is the leading cause of morbidity and mortality, which affects many parts of the body, including the brain, extremities, and internal organs. The incidence of life-threatening complications, such as systemic inflammatory response syndrome (SIRS) and acute lung injury (ALI), in severely injured trauma patients remains between 30% and 50% [1, 2]. SIRS induced by severe trauma or other injuries is a clinical syndrome initiated by dysregulation of inflammation, which could lead to various tissue injuries culminating in multiple organ dysfunction/failure syndrome (MODS/MOF).

Bone marrow-derived mesenchymal stem cells (BMSCs), also referred to as marrow stromal cells, are a multipotential lineage characterized by the capacity for extracorporeal expansion and the ability to differentiate into bone, cartilage, and adipose tissues. BMSCs appear to function as potent immunomodulators. Numerous studies indicated that BMSCs can be an effective modality for cell-based immunomodulatory therapies in various diseases, such as sepsis, endotoxemia, diabetes, and lung injury [3–9], especially for reducing of inflammation [4, 5, 10]. Several clinical trials assessing the efficacy of BMSCs in immune-mediated diseases are currently underway. However, how BMSCs attenuate inflammatory reaction is poorly understood.

Quick and efficient response to microbial infections is driven by recognition of molecules broadly shared by a variety of pathogens, which are distinguishable from host molecules named pathogen associated molecular patterns (PAMPs). Pattern-recognition receptors (PRRs) that contain membrane-bound PRRs, including toll-like receptors (TLRs) and other cytoplasmic proteins, recognize the PAMPs [10]. Upon binding with the PAMPs, TLR2 and TLR4 then activate NF-*κ*B and subsequently promote cytokine synthesis, which include IL-1, IL-6, and TNF-*α* [11, 12]. The network of TLRs and PRRs mediates the response of BMSCs to inflammatory stimuli, such as LPS [13, 14]. However, how TLRs modulate the BMSC activity is not clearly elucidated. Since TLR2 and TLR4 response to acute otitis through activation of NF-*κ*B [15], we hypothesized that BMSCs ameliorate the inflammation damage of alveolar macrophages and HUVECs through the inhibition of TLR2 and TLR4 mediated NF-*κ*B pathways.

## 2. Materials and Methods

### 2.1. Cell Culture

Alveolar macrophages and HUVECs were purchased from Chi Scientific, Inc. (China). The cells were cultured in RPMI 1640 medium (Sigma Chemical Co., St. Louis, MO) supplemented with 10% fetal bovine serum (FBS) (Gibco, Carlsbad, CA, USA) and 1% penicillin/streptomycin (Solarbio, Beijing, China) at 37°C in a humidified atmosphere of 5% CO_2_. BMSCs obtained from Cyagen Biosciences Inc. (Suzhou, China) were cultured in 10% FBS-DMEM-LG (Gibco, Carlsbad, CA, USA). The surface markers for BMSCs differentiation of adipocytic, osteogenic, and chondrogenic lineages were characterized as previously reported [16]. Only passages 3–7 of the cells were used for experiments.

### 2.2. Endothelial Permeability Assay

Monolayer permeability was quantitated by spectrophotometric measurement of the flux of Evans blue-albumin across HUVECs as described previously [17]. HUVECs (1 × 10^5^) were seeded in 24-well Transwell inserts (5 *μ*m pore size, Corning Incorporated, NY, USA) and cultured with 10% FBS DMEM overnight and then changed with serum-free medium for another 24 hours. The various numbers of BMSCs were seeded at the bottom of the same 24-well plate with serum-free media. LPS was added to the Transwell inserts at a final concentration of 100 ng/mL for different time points (1, 3, 6, 12, and 24 h). After that, the BMSCs were digested by trypsin-EDTA solution to terminate the effects of BMSCs and 100 *μ*L Hank's solution containing Evans blue- (EB-) conjugated bovine serum albumin (final concentration 0.67 mg/mL) was added to the Transwell inserts, while 600 *μ*L 4% BSA was added to the lower plate chamber. After incubation at 37°C for 1 hour, 100 *μ*L BSA solution from the lower chamber was harvested and measured for the absorbance at 620 nm. The HUVECs permeability capacity was calculated as follows: Evans blue dye-labeled albumin (EB-albumin) leak  rate = OD620_leaked  EB-albumin_/OD620_total  EB-albumin_ × 100%.

### 2.3. Alveolar Macrophages/HUVECs-BMSC Coculture Experiments

Alveolar macrophages or HUVECs (1 × 10^6^/well) were seeded in a 6-well plate, after 24-hour incubation, BMSCs (2 × 10^5^/well) were seeded in the 6-well Transwell inserts (5 *μ*m pore, Corning Incorporated, NY, USA) at the indicated time points (1, 3, 6, 12, and 24 h). LPS (100 ng/mL) was then added to the lower chamber for 1 h. Alveolar macrophages or HUVECs lysate was collected for RNA and protein analyses, and the cell-free supernatants were collected for ELISA detection.

### 2.4. Western Blot Analysis

Alveolar macrophages/HUVECs were lysed in the RIPA buffer (Thermo Scientific, Rockford, USA) supplemented with 1% PMSF and 1% protein phosphatase inhibitor mixture (P1260, Applygen, Beijing, China). The concentration of the proteins was measured with the Bicinchoninic Acid Kit (Thermo Scientific, Rockford, USA). Rabbit anti-TLR2, TLR4, and phosphorylated p65 unit of NF-*κ*B antibodies were obtained from Cell Signaling Technology. Horseradish peroxidase- (HRP-) conjugated goat anti-rabbit antibodies were purchased from Bio-Rad. ECL-Plus Chemiluminescent Reagent (Thermo Scientific, Rockford, USA) was used to visualize the specific proteins. Relative concentration of proteins was quantitated using the Image J Software (National Institutes of Health, Bethesda, USA).

### 2.5. Gene Expression Analysis

Total RNA was isolated from cells using the TRIzol RNA Isolation Reagents (Life Technologies). The first-strand cDNAs were converted using the SuperScript III reverse transcriptase (Invitrogen, Carlsbad, CA) according to the manufacturer's protocols. Real-time RT-PCR analysis was performed on ABI 7500 Sequence Detection System using the UltraSYBR Mixture (CW Bio Co. Ltd, Beijing, China). Transcript expression was normalized with the glyceraldehyde-3-phosphate dehydrogenase (GAPDH) housekeeping gene. The PCR primer sequences are GAPDH forward 5′-TGGAGTCTACTGGCGTCTT-3′, reverse 5′-TGTCATATTTCTCGTG GTTCA-3′; TLR4 forward 5′-CTGCATAGAGGTAGTTCCT-3′, reverse 5′-TCCAGCC ACTGAAGTTCTGA-3′; TLR2 forward 5′-GGAGACTCTGGAAGC ATG-3′, reverse 5′-GCATCCT GAAGCCTGTG-3′.

### 2.6. ELISA Analysis

The supernatants collected from coculture system were centrifuged for 10 minutes at 1000 g (4°C) to remove cell debris. The levels of TNF-*α* and IL10 were measured using commercially available enzyme-linked immunosorbent assay (ELISA) kits (BD Biosciences, San Diego, CA, USA) according to the instructions provided by the manufacturer.

### 2.7. Statistical Analysis

SPSS 17.0 software was used to perform the statistical analysis and values are presented as the means ± standard deviations (SD); all experiments were repeated at least three times. The one-way ANOVA was used to determine the significance of differences between various groups. *P* < 0.05 was considered to be statistically significant.

## 3. Results

### 3.1. BMSCs Inhibit LPS to Induce Hyperpermeability in HUVECs

**Figure 1:**
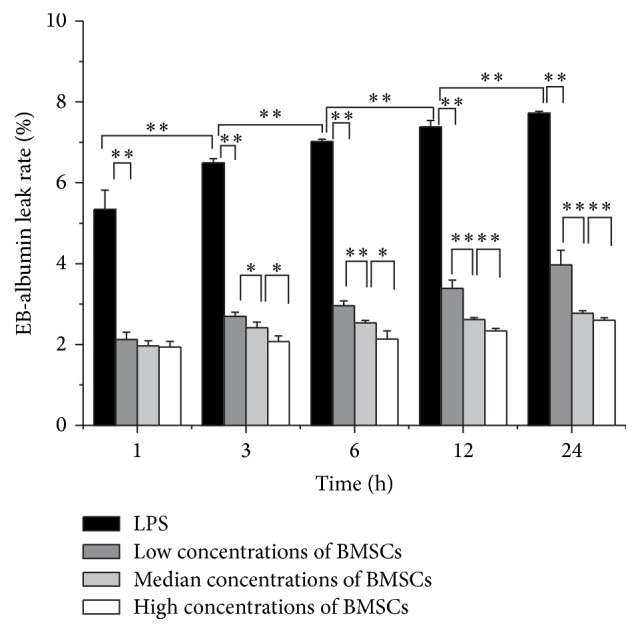
Effect of BMSCs on the barrier permeability of HUVECs. HUVECs were stimulated by LPS at a dosage of 100 ng/mL (black bars). In addition, HUVECs were cocultured with indicated concentrations of BMSCs: low (1 × 10^3^ cells/mL, dark grey bars), median (1 × 10^4^ cells/mL, light grey bars), and high (1 × 10^5^ cells/mL, white bars) for 1, 3, 6, 12, and 24 h followed by measuring permeability as described in Section 2. All results are means ± SD of at least three different experiments. ^*∗*^ *P* < 0.05; ^*∗∗*^ *P* < 0.01.

To assess how BMSCs affected HUVECs permeability induced by LPS, HUVECs with or without coculture with BMSCs were stimulated by LPS for the indicated times. Without coculture with BMSCs, the EB-albumin leaking rate ranged from 5.35% (at 1 h) to 7.72% (at 24 h) in the LPS group (Figure 1). Compared with the LPS group, the EB-albumin leaking rates in the BMSC coculture group were reduced in a BMSC cell number- and time-dependent manner. The EB-albumin leaking rate in the 1 × 10^4^/mL BMSCs group was decreased 3 hours after coculture. The effects were more significant after coculture for 6 hours to 24 hours. The effects were less significant in the group with 1 × 10^3^/mL BMSCs and more significant in the group with 1 × 10^5^/mL BMSCs (Figure 1).

### 3.2. BMSCs Attenuate LPS-Induced Expression of TLR2, TLR4, and p65 in Alveolar Macrophages and HUVECs

**Figure 2:**
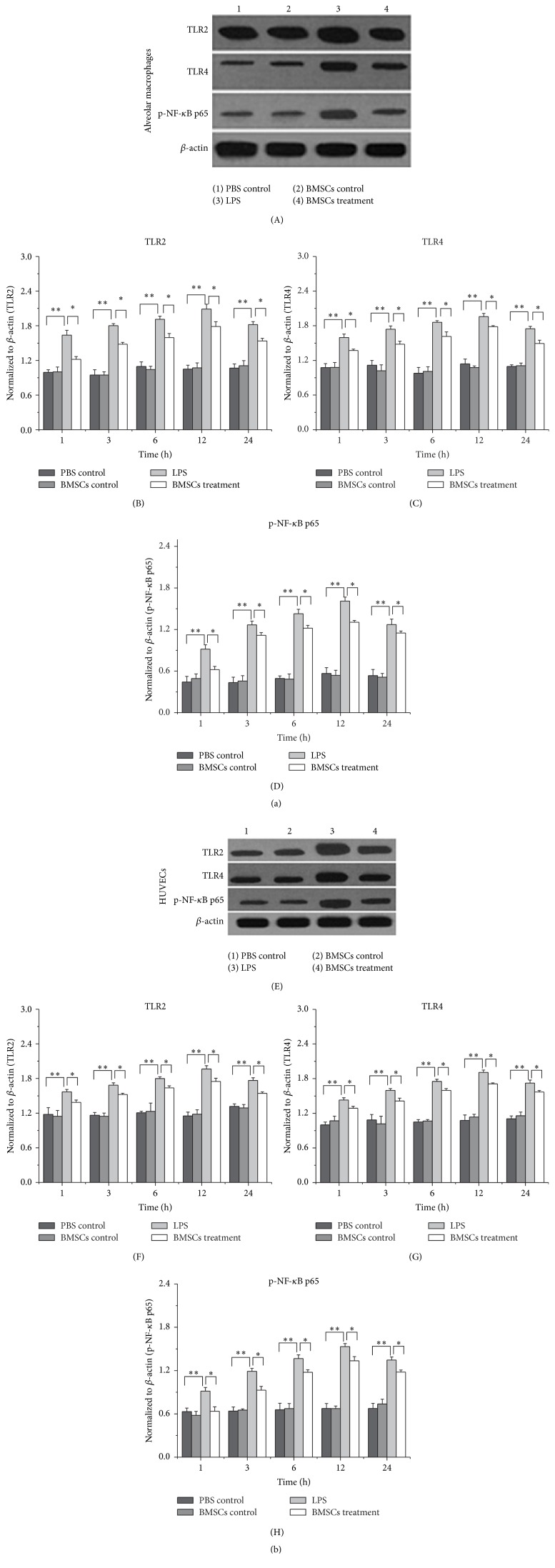
Coculture with BMSCs attenuates the activity of LPS to induce expressions of TLR2, TLR4, and p65 in alveolar macrophages (a) and HUVECs (b) at the protein level. The total proteins were extracted from the cultured alveolar macrophages and HUVECs treated (1) PBS control, (2) BMSCs, (3) LPS, and (4) BMSCs and LPS. The expressions of TLR2, TLR4, and p-NF-*κ*B p65 were determined by Western blot. The density of specific bands was quantitated. Data are expressed as the mean ± SD from three experiments. ^*∗*^ *P* < 0.05, ^*∗∗*^ *P* < 0.01.

**Figure 3:**
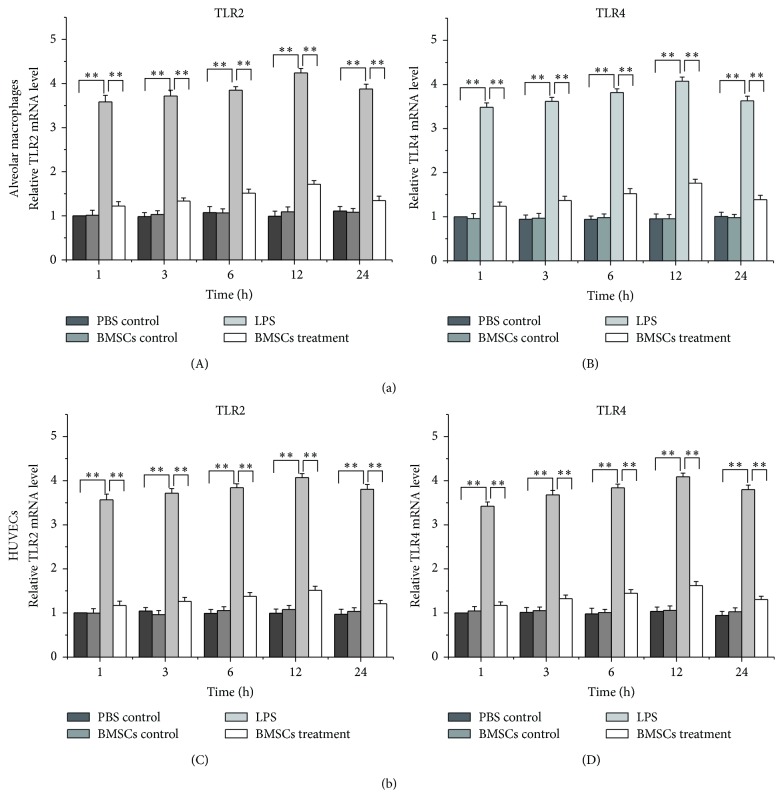
Coculture with BMSCs attenuates the activity of LPS to induce expressions of TLR2 and TLR4 in alveolar macrophages (a) and HUVECs (b) at the mRNA level. The total RNAs were extracted from the cultured alveolar macrophages and HUVECs treated (1) PBS control, (2) BMSCs, (3) LPS, and (4) BMSCs and LPS. The expressions of TLR2 and TLR4 were determined by real-time RT-PCR. Data are expressed as the mean ± SD from three experiments. ^*∗*^ *P* < 0.05, ^*∗∗*^ *P* < 0.01.

To determine whether LPS stimulated expression of TLR2, TLR4, and p65 in macrophages and HUVECs was affected by BMSCs, alveolar macrophages and HUVECs cells were cocultured with BMSCs for the indicated time followed by LPS treatment. Western blot (Figure 2) and real-time RT-PCR analyses (Figure 3) showed that LPS administration increased expression of TLR2, TLR4, and p65 within 1 hour after the treatment. The effect persisted even until 24 hours after the treatment. However, the activity of LPS was attenuated by coculture with BMSCs in both alveolar macrophages and HUVECs (Figures 2 and 3).

### 3.3. BMSCs Suppress Alveolar Macrophages and HUVECs to Release IL10 and TNF-*α* in Response to LPS Stimulation

**Figure 4:**
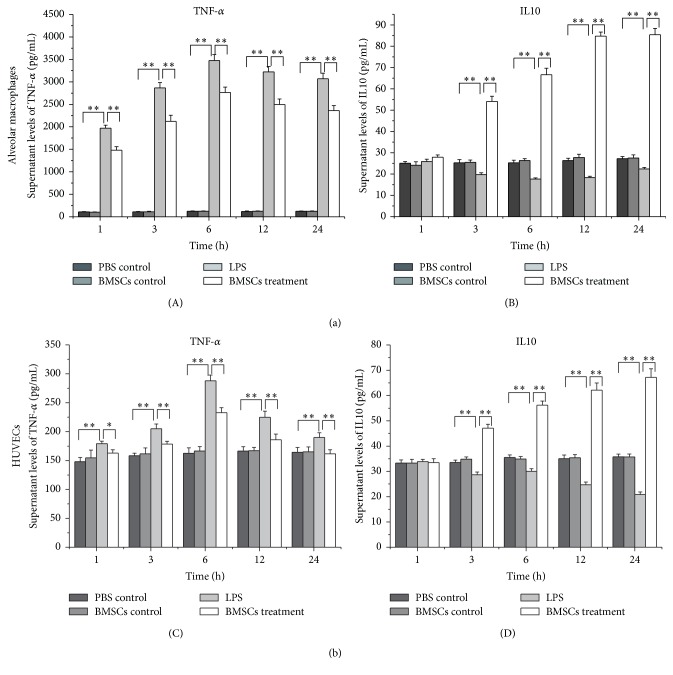
Coculture with BMSCs attenuates the activity of LPS to induce release of cytokines in alveolar macrophages (a) and HUVECs (b). Alveolar macrophages and HUVECs with or without coculture with BMSCs were treated with LPS or PBS for 1, 3, 6, 12, and 24 hours. The cell culture supernatant was then collected for cytokine analysis by ELISA. Data are expressed as the mean ± SD from three experiments. ^*∗*^ *P* < 0.05, ^*∗∗*^ *P* < 0.01.

To determine whether BMSCs regulated the release of TNF-*α* and IL10 from alveolar macrophages and HUVECs in response to LPS stimulation, the conditioned medium was collected from the coculture system and the concentrations of IL10 and TNF-*α* were assessed by ELISA. It was clear that concentration of TNF-*α* in the medium was increased after LPS stimulation within 1 hour and the effects lasted for 24 hours. However, the increases were blunted by coculture with BMSCs. In contrast, IL10 was reduced by LPS stimulation, while coculture with BMSCs increased medium IL10 production. The results indicate that BMSCs suppress LPS-induced inflammation by decreasing TNF-*α* and increasing IL10 releases (Figure 4).

## 4. Discussion

BMSCs treatments have been shown to reduce inflammatory response in human and animal models in response to injury. However, the detailed mechanism underlying this effect is not clear. Here, we reported that BMSCs alleviated HUVEC damage induced by LPS. The results also showed that BMSCs restricted the inflammatory responses of HUVECs and alveolar macrophage stimulated by LPS and therefore protected HUVECs from damage induced by the endotoxin. The data indicate that attenuating LPS-induced TNF-*α* pathway activation in HUVECs and macrophage by BMSCs contributes to the protection of HUVECs from endotoxin-induced damage by BMSCs.

Our data demonstrated that LPS increased the permeability of HUVECs monolayers in a time-dependent manner. Although the extent of the activity was lower than that after coculture for a longer time, the protection effects were detectable within one hour. The underlying mechanism of the protection is not clear. Since BMSCs and HUVECs were cultured separating by the Transwell membrane, the effects of BMSCs must be mediated by soluble factors released from BMSCs. However, we did not rule out the possibility that BMSCs sequestrated LPS and therefore protected the endothelial cells. Future efforts are needed to identify these beneficial secretory factors from BMSCs. Although LPS still induced HUVECs damage in the coculture, the EB-albumin leaks were reduced compared with the group without coculture with BMSCs. This suggests that BMSCs attenuate the damage. We observed that the protective effect of BMSCs on HUVEC was cell number-dependent. High concentrations of BMSCs were more effective than low concentrations of BMSCs. It appeared that the BMSCs released sufficient protective molecules to protect endothelial cells within one hour. Therefore, immediately administration of BMSCs to SIRS patients shall have beneficial effects. Furthermore, the results were in line with previous reports that LPS leads to persistent damage of HUVECs [18, 19]. However, it was different from the report that the damage of HUVEC by LPS is not time-dependent [17]. The discrepancy is likely due to different culture time. We cultured the cells for 24 hours to reach the confluence, followed by serum starvation for 24 hours and LPS treatment. This method is different from the published one where the cells were cultured for 4 days prior to the experiments. Further efforts are needed to clarify this discrepancy.

TLRs play a pivotal role in defense against invading pathogens through recognizing PAMPs. It is well documented that cells use TLR2 and TLR4 to recognize PAMPs expressed by bacteria. Activation of TLR2 and TLR4 leads to activation of MAPK and NF-*κ*B pathways and production of inflammatory cytokines, including IL-1, IL-6, and TNF-*α* [20–22]. In this study, we showed that LPS promoted expression of TLR2 and TLR4 in alveolar macrophages and HUVECs. However, how expression of TLR2 expression was increased by LPS is unknown and deserves future investigations. TNF-*α* and IL10 expressions were also affected by LPS. Furthermore, the LPS effects were diminished when the HUVECs and macrophages were cultured with BMSCs. TNF-*α* is a potent inflammatory factor and IL10 is a key anti-inflammatory cytokine. Downregulation of TNF-*α* and upregulation of IL10 in HUVECs and macrophage indicate that the inflammatory reactions were suppressed. The results suggest that BMSCs have the ability to alleviate pathogen-induced inflammation damage in HUVECs and macrophages.

In conclusion, the results showed that BMSCs attenuated the activity of LPS on the inflammatory reaction of HUVECs and alveolar macrophages through inhibiting upregulation of TLR2, TLR4, and p65 expression, as well as downregulating TNF-*α* release and upregulating IL10 release.

## Abbreviations

BMSC: Bone marrow mesenchymal stem cells
LPS: Lipopolysaccharide
TLR: Toll-like receptor
SIRS: Systemic inflammatory response syndrome
MODS: Multiple organ dysfunction syndrome
PAMP: Pathogen associated molecular patterns.
